# Supplementary material for: Structural Dissection of the First Events Following Membrane Binding of the Islet Amyloid Polypeptide
Source: Front Mol Biosci. 2022 Mar 15;9:849979. doi: 10.3389/fmolb.2022.849979 (PMC8965455; doi:10.3389/fmolb.2022.849979)
Supplement: Supplementary file 1 [file DataSheet1.PDF]

Supplementary Material

Structural dissection of the first events following membrane binding of the islet amyloid polypeptide

Lucie Khemtemourian[1,*], Hebah Fatafta[2,3], Benoit Davion[1], Sophie Lecomte[1], Sabine Castano[1] and Birgit Strodel[2,3,4,*]

[1] Université de Bordeaux, CNRS, Bordeaux INP, CBMN, UMR 5248, F-33600 Pessac, France.

[2] Institute of Biological Information Processing: Structural Biochemistry, Forschungszentrum Jülich, 52428 Jülich, Germany.

[3] JuStruct, Jülich Center for Structural Biology, Forschungszentrum Jülich, 52428 Jülich, Germany

[4] Institute of Theoretical and Computational Chemistry, Heinrich Heine University Düsseldorf, 40225 Düsseldorf, Germany.

* Corresponding Authors: lucie.khemtemourian@u-bordeaux.fr, b.strodel@fz-juelich.de

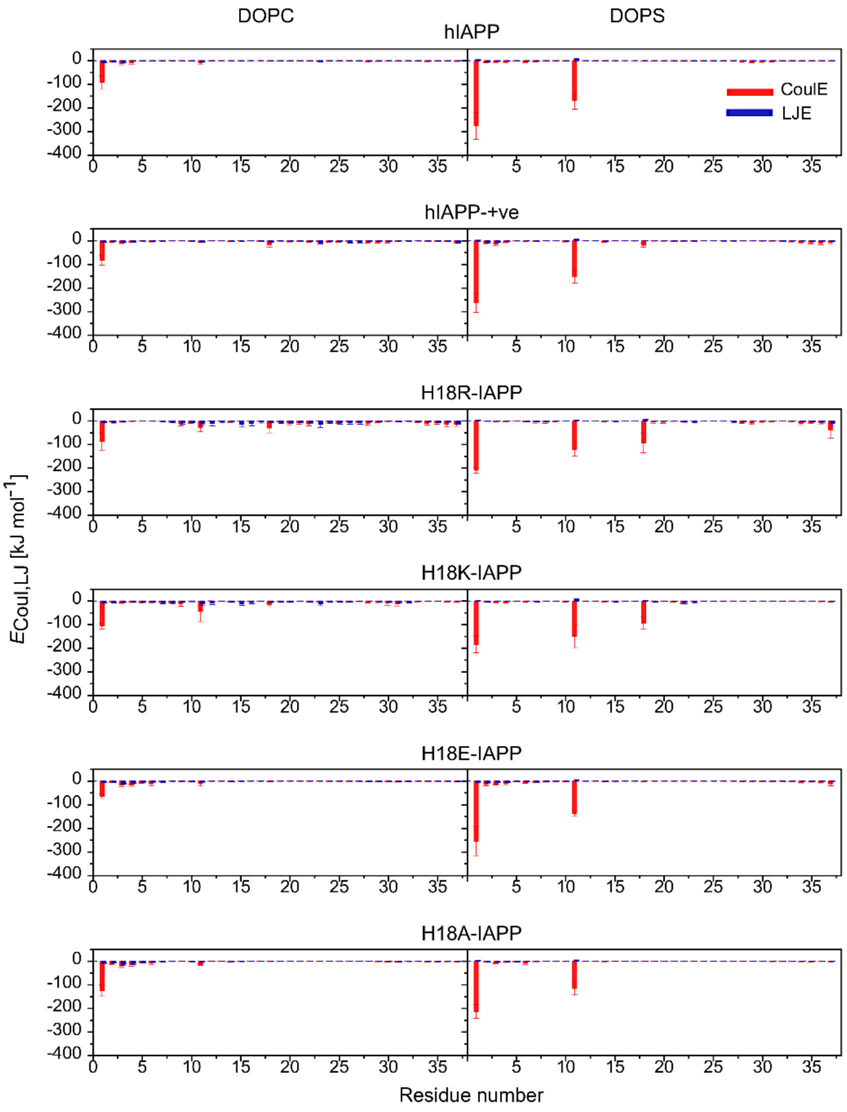

Figure S1: The average interaction energies (and standard error) of IAPP interacting with DOPC (left) and DOPS (right) lipids. Electrostatic and Lennard-Jones energies are shown in red and blue, respectively. Negative energies indicate attractive forces, positive energies correspond to repulsion.

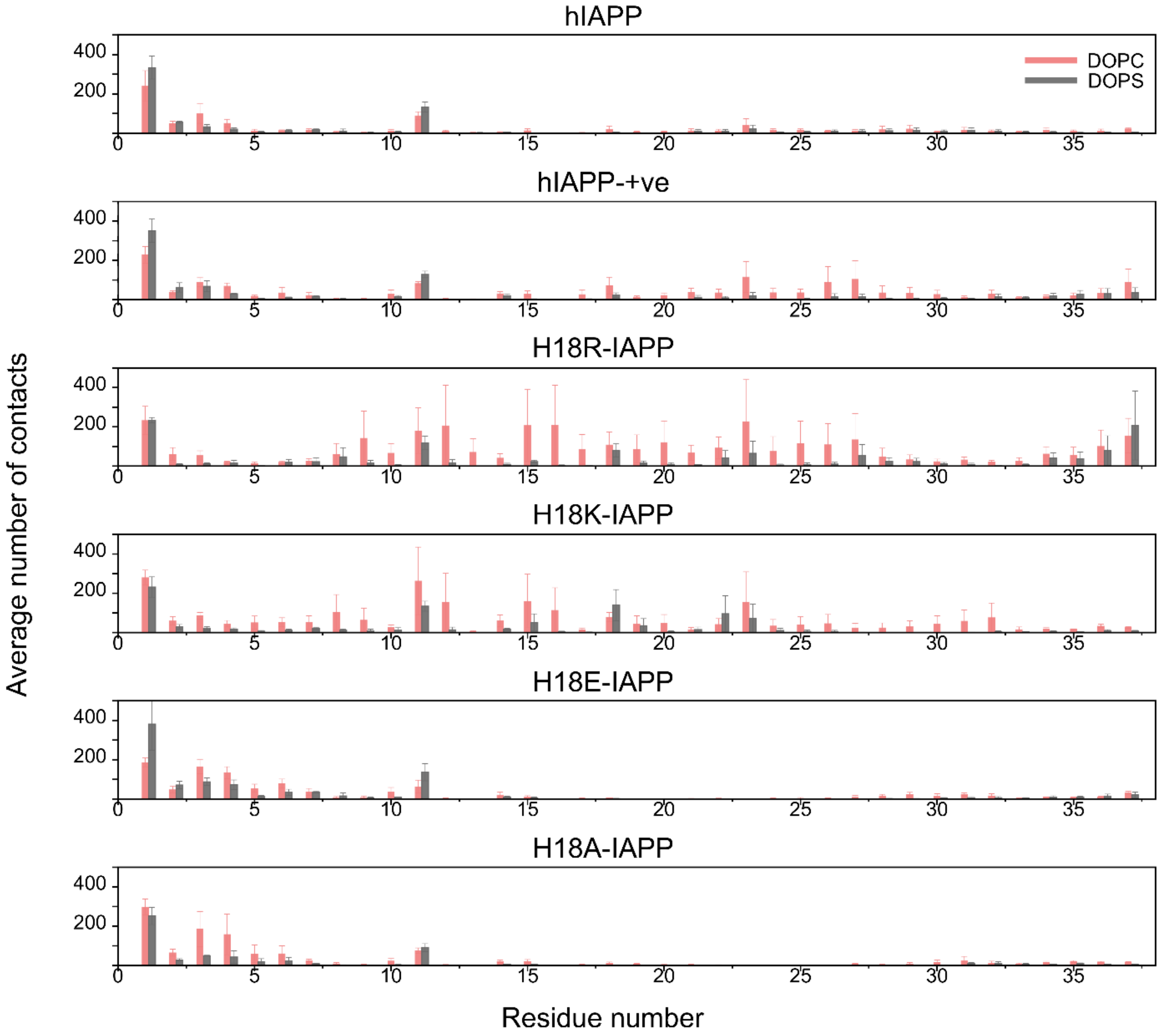

Figure S2: The average number of IAPP-lipid contacts (and standard error) for DOPC (pink) and DOPS (gray) lipids.

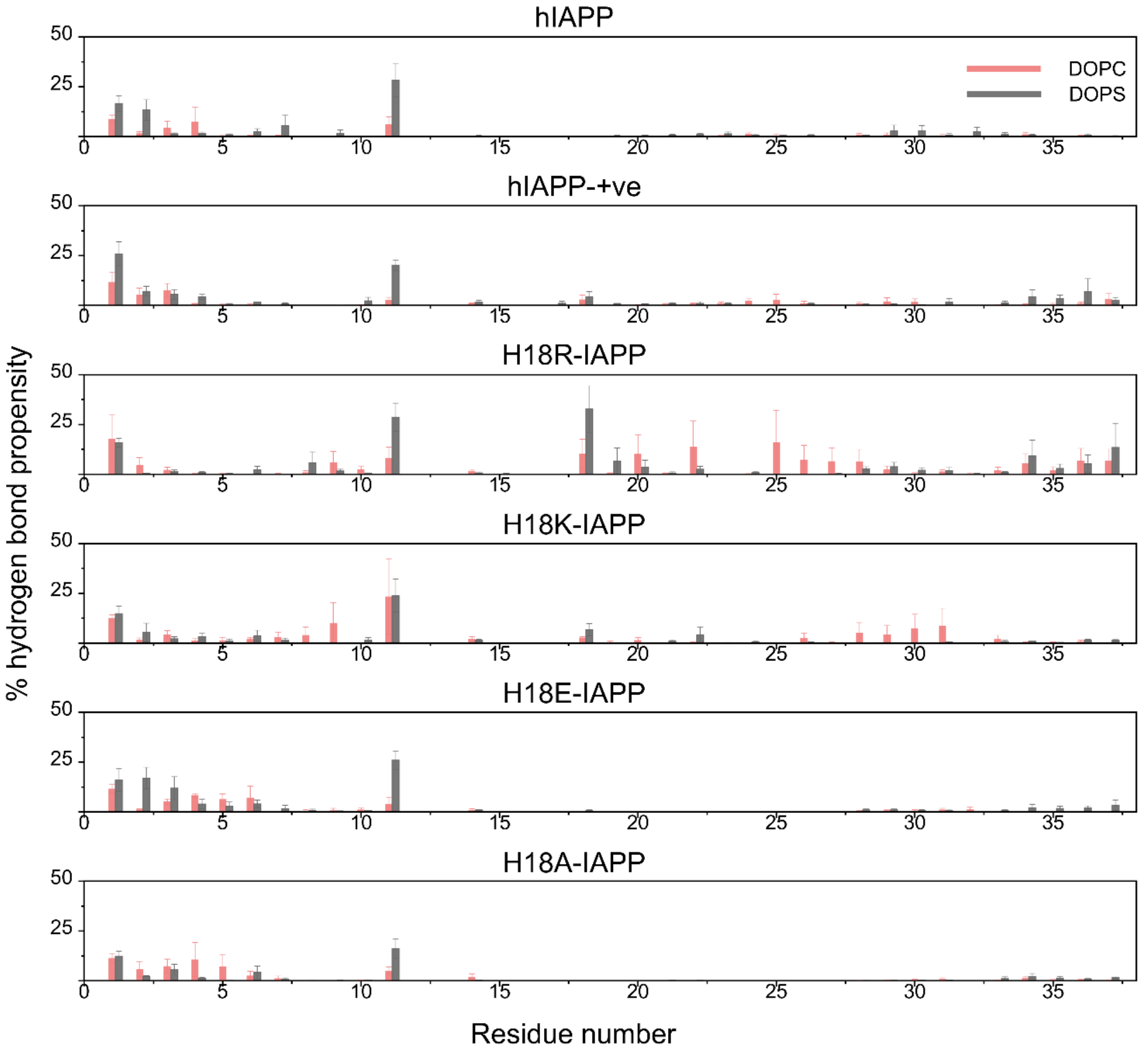

Figure S3: The average hydrogen bond propensity (and standard error) between IAPP and DOPC (pink) and DOPS (gray) lipids.

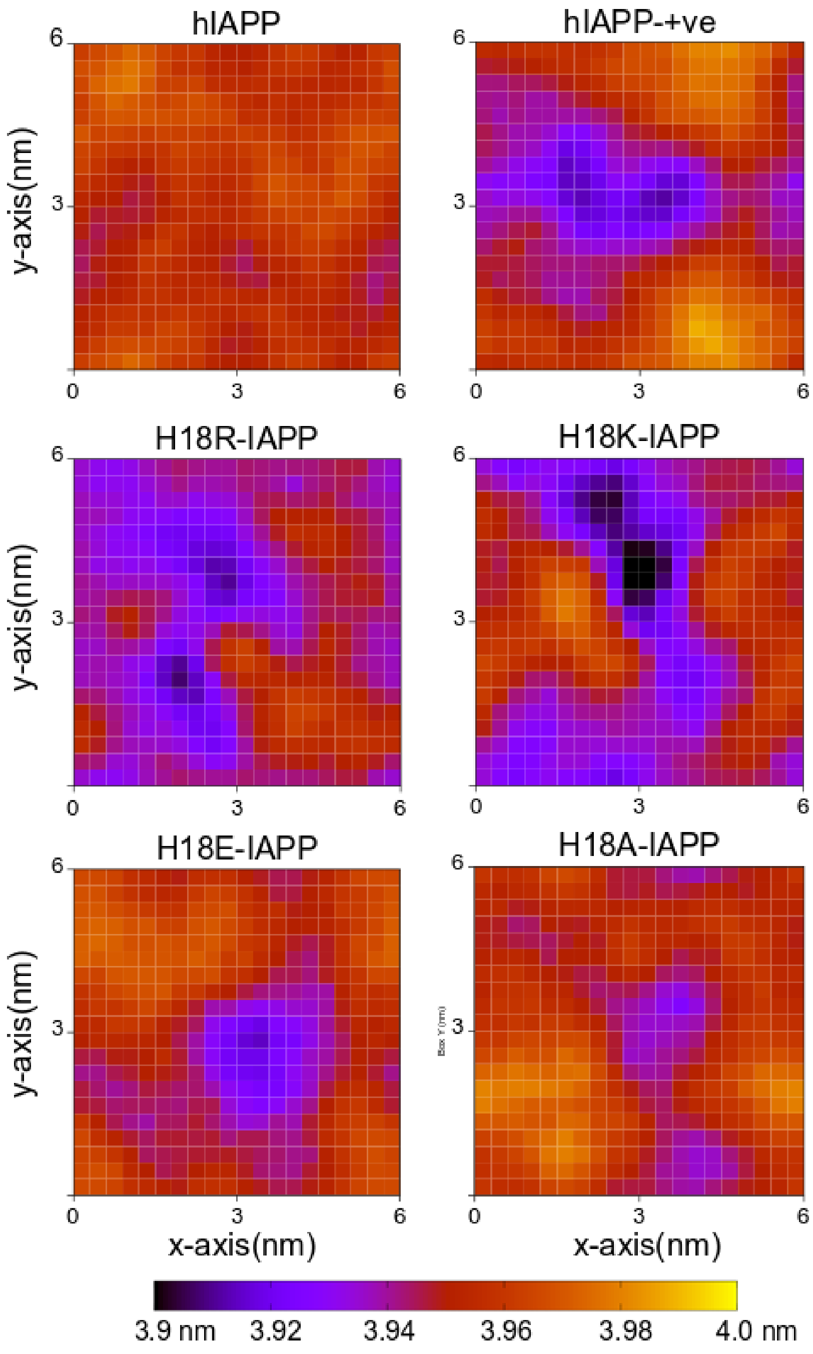

Figure S4: Average bilayer thickness calculated for the MD frames where the peptide is within 0.5 nm of the membrane. The *x*- and the y-axes represent the unit cell dimension in nm. The color bar shows the thickness range in nm.

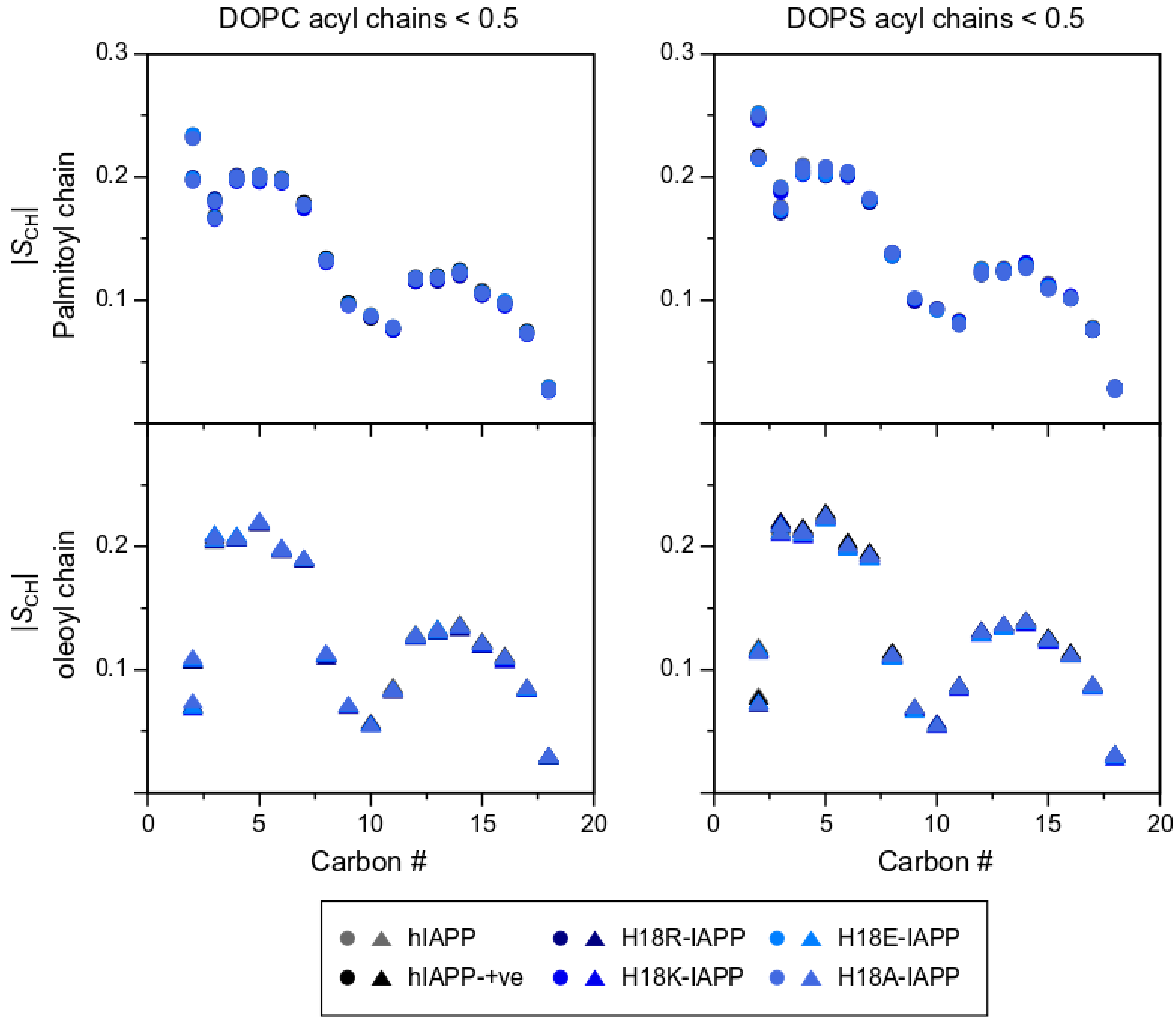

Figure S5: Average order parameters of the acyl chains (top: palmitoyol chains; bottom: olyeol chains) of DOPC (left) and DOPS (right) lipids.
